# Assessing the Impact of a Rapid Response Team on Code Blue Incidents and Hospital Mortality Rate: Evidence From a Tertiary Care Hospital in India

**DOI:** 10.7759/cureus.101923

**Published:** 2026-01-20

**Authors:** Leo Tom, Rajeesh R Pillai, Gautham Rajan, Kanniyan Binub

**Affiliations:** 1 Internal Medicine, K S Hegde Medical Academy, Mangaluru, IND; 2 Internal Medicine, Malabar Medical College and Research Centre, Kozhikode, IND; 3 Emergency Medicine, Malabar Medical College and Research Centre, Kozhikode, IND; 4 Critical Care Medicine, Malabar Medical College and Research Centre, Kozhikode, IND; 5 Community Medicine, Malabar Medical College and Research Centre, Kozhikode, IND

**Keywords:** code blue simulation, emergency response plan, in hospital cardiac arrest, in hospital mortality, medical intensive care unit (micu), patient outcome research, quality improvement projects, rapid response team (rrt)

## Abstract

Background: Rapid Response Teams (RRTs) are designed and implemented to identify and respond to early signs of clinical deterioration, to prevent in-hospital adverse events, particularly cardiopulmonary arrests. This multidisciplinary team aims to improve patient outcomes and reduce hospital mortality. Despite their widespread adoption in high-resource settings, evidence on the efficacy of RRTs in resource-limited environments remains limited.

Objective: The objective of this study was to evaluate the impact of implementing an RRT on patient outcomes in a tertiary care hospital in South India.

Methods: This study compared data from six months before (November 2022 to April 2023) and six months after (November 2023 to April 2024) starting RRT. Event rates were analyzed using unadjusted and adjusted Poisson regression for all three outcomes. In-hospital mortality was additionally assessed using logistic regression and interrupted time series analysis to evaluate trends over time.

Results: Among 24,568 hospital admissions, in-hospital mortality decreased from 3.7% to 2.9% post-RRT implementation (absolute difference: 0.71%; p = 0.002). Code Blue events declined from 2.3 to 1.5 per 1,000 admissions, and unplanned MICU transfers remained stable; however, these changes were not statistically significant. Unadjusted Poisson analysis showed a significant reduction in mortality (rate ratio: 0.81; 95% CI: 0.70-0.93; p = 0.003), though adjusted models showed no significant effects. Interrupted time series analysis demonstrated a significant month-on-month decline in mortality post-intervention (OR: 0.91; 95% CI: 0.83-0.99; p = 0.021), suggesting a gradual impact over time.

Conclusion: RRT implementation was associated with a significant reduction in in-hospital mortality, with evidence of a sustained downward trend over time. Effects on Code Blue events and MICU transfers were not statistically significant, warranting further evaluation in similar settings.

## Introduction

In-hospital cardiac arrests remain a major source of mortality in acute care settings, with approximately 290,000 cases reported annually in the United States alone [1]. Numerous studies have shown that many of these events are preceded by signs of physiological deterioration, offering a potential window for early detection and intervention. While 13.4% of patients exhibit at least one critically abnormal vital sign prior to cardiac arrest, nearly 60% present with at least one abnormal vital parameter in the four hours preceding the event [2]. Identifying such patients in time and intervening effectively can significantly reduce the incidence of cardiopulmonary arrest and its associated mortality [3].

To address this early warning window, the concept of Rapid Response Teams (RRTs) has been widely adopted over the past two decades in hospital systems worldwide [4]. The first Medical Emergency Team (MET), a precursor to modern RRTs, was introduced at Liverpool Hospital in Australia in 1995 [5]. The movement gained significant traction following the Institute for Healthcare Improvement (IHI) 100,000 Lives Campaign in 2004, which emphasized timely identification and intervention to reduce preventable inpatient morbidity and mortality [6].

Despite widespread adoption across high-income countries, the effectiveness of RRTs in improving hospital outcomes remains a subject of debate, with studies showing inconsistent impact on in-hospital cardiac arrests, all-cause hospital mortality, and unplanned intensive care unit (ICU) transfers [7-14]. For instance, the Medical Emergency Response and Intervention Trial (MERIT), one of the earliest and largest cluster-randomized evaluations, did not find a significant reduction in cardiac arrests or mortality following the implementation of a medical emergency team [9]. Similarly, a systematic review and meta-analysis by Chan et al. (2010) reported decreased rates of cardiopulmonary arrest but no consistent improvement in mortality [3]. More recently, a commentary by Edelson (2010) emphasized that the effectiveness of RRTs may be limited not only by clinical variability but also by system-level weaknesses, such as inconsistent activation criteria, delays in recognition, and poor integration with existing workflows, highlighting that measurable benefits may only emerge when these foundational issues are addressed [15]. Collectively, these results have prompted ongoing discussion regarding the foundational premise of RRTs, particularly in resource-constrained or high-acuity settings.

RRTs are fundamentally different from traditional emergency response systems. While ‘Code Blue’ is typically activated during cardiopulmonary arrest requiring immediate resuscitation, RRTs are activated earlier, when patients first show signs of clinical deterioration, such as altered mental status, hypoxia, or abnormal vital signs. Through proactive monitoring and multidisciplinary intervention, RRTs aim to prevent clinical deterioration from progressing to critical events.

In India, the implementation of RRTs is still in its nascent stages. Challenges such as limited healthcare resources, high patient-to-nurse ratios, and heterogeneity in hospital infrastructure complicate large-scale adoption. Moreover, local evidence on the effectiveness of RRTs in Indian hospitals is extremely limited. As such, there is a pressing need for context-specific research to evaluate whether RRTs can be effective in improving outcomes under such constraints. Kerala, a state in South India, in particular, is an interesting case for study. Despite being recognized for its relatively advanced healthcare indicators and high literacy rates, public and private hospitals in the state still face operational and capacity-related challenges that may influence RRT effectiveness.

The aim of this study was to estimate the impact of RRT implementation on key hospital outcomes, including in-hospital mortality, unplanned transfers to the medical intensive care unit (MICU), and Code Blue activations, in a tertiary care teaching hospital in Kerala. By comparing data from two equivalent six-month periods before and after implementation, this study seeks to contribute to the limited but growing body of evidence on the utility of RRTs in low-resource and high-burden settings.

## Materials and methods

This retrospective observational study was conducted at Malabar Medical College Hospital and Research Centre, an 890-bedded tertiary care teaching hospital in Kerala, India. The study aimed to evaluate the impact of implementing an RRT on key inpatient outcomes. The study was approved by the Malabar Medical College Hospital and Research Centre Institutional Ethics Committee (MMCH&RC/IEC/2024/Aug/63).

Study design and population

The RRT system was formally launched in July 2023 to provide a structured response to acute clinical deterioration in hospital wards. The initial post-launch period involved phased implementation, including staff training, protocol familiarisation, and progressive integration into routine ward workflows. To minimise exposure misclassification and contamination between study phases, this implementation period (July-October 2023) was excluded from the analysis. Accordingly, the post-RRT phase was defined as beginning in November 2023, when the RRT was fully operational and consistently implemented across hospital wards.

The analysis covered two equivalent six-month periods: the pre-RRT phase from November 2022 to April 2023, and the post-RRT phase from November 2023 to April 2024. These periods were separated by one calendar year and matched to identical months to minimise potential seasonal confounding. All hospital admissions during these periods were included, with a total of 24,568 admissions considered for analysis: 11,150 in the pre-RRT phase and 13,418 in the post-RRT phase. No exclusion criteria were applied, except for cardiac arrest events, which continued to be managed through a separate Code Blue protocol.

The RRT was formally developed and implemented as a hospital-wide policy with predefined protocols for activation, response, and escalation. The policy was instituted through the hospital’s quality and patient safety framework and remained in effect from the time of implementation onward. The RRT at the hospital consisted of an emergency medicine physician, an on-call specialist (from Internal Medicine, General Surgery, or Pediatrics), a critical care nurse, a resident doctor from the relevant department, and a nursing supervisor. For Code Blue activations, a critical care physician was additionally present as part of the resuscitation team. It could be activated by any hospital staff member, including nurses, doctors, or allied health workers. Once activated, the team was expected to respond to the patient's bedside within five minutes. Prior to implementation, RRT members underwent structured training and simulation-based drills to ensure readiness, clarify roles, and familiarise staff with activation criteria and response workflows. Standardized activation criteria for the RRT were developed and circulated to all clinical departments. These criteria were designed to assist frontline staff in promptly identifying patients showing early signs of physiological deterioration. The complete list of activation indicators is presented in Table 1. Any hospital staff member-nurse, doctor, or allied professional-could activate the RRT upon recognition of these signs, with the expectation that the team would respond within five minutes of activation.

**Table 1:** Indicators for alerting the Rapid Response Team SpO2: peripheral oxygen saturation

Indicators
1. Heart rate < 40 beats per minute or > 130 beats per minute
2. Respiratory rate < 10 breaths per minute and > 30 breaths per minute
3. Chest pain or any chest discomfort
4. Unexplained altered mental status
5. Acute fall in saturation (SpO2 <90% on supplemental oxygen)
6. Acute decrease in urine output (< 100 ml in four hours)
7. Systolic blood pressure < 90 mmHg
8. Agitated patient
9. New onset or prolonged seizures
10. Any abnormal bleeding
11. Nursing/Family concern about patient status

Upon arrival, the RRT conducted a rapid clinical assessment using the ABCD (Airway, Breathing, Circulation, Disability) approach and initiated necessary interventions. Depending on the clinical scenario, the patient was either stabilized in the ward or transferred to the MICU. Coordination with the treating team was maintained throughout the episode. All RRT activations were documented using a standardized, single-page RRT activation form capturing patient details, clinical parameters, and actions taken (see Appendices).

Data collection and variables

Data for the study were collected retrospectively from multiple institutional sources, including the Medical Records Department, RRT activation logs, the Code Blue activation registry, and MICU admission records, all of which are routinely maintained and audited by hospital nursing and management teams as part of standard quality assurance processes. A structured data extraction form was developed and used by trained researchers. To ensure data integrity, double data entry was independently performed by two individuals, and discrepancies were resolved through cross-verification. Importantly, there were no changes in ICU bed capacity, staffing ratios, or patient monitoring systems during the study period that could confound the outcomes.

The primary outcome assessed was in-hospital mortality, expressed both as a percentage of total admissions and as a rate per 1,000 hospital admissions. Secondary outcomes included the number of Code Blue events and the number of unplanned transfers to the MICU, expressed as a rate per 1000 hospital admissions. A Code Blue event was defined as the activation of the hospital emergency response system for a confirmed or suspected cardiac arrest, and was limited to ward-based cardiac arrests, with arrests occurring in the ICU or emergency department excluded, consistent with institutional Code Blue registry definitions. Unplanned MICU transfers were defined as transfers resulting from acute clinical deterioration and occurring subsequent to RRT activation. These were distinguished from planned MICU transfers, which were pre-emptively identified during routine ward rounds by the treating physician.

Statistical analysis

Descriptive statistics were used to summarise the data across the pre- and post-RRT periods. Event rates between the two phases were compared using Poisson rate tests, with total hospital admissions used as the exposure variable. For in-hospital mortality, a two-proportion z-test was performed to compare absolute differences in mortality percentages between periods.

To adjust for time trends and potential confounding, multivariable Poisson regression models were constructed for each outcome, with time specified as a discrete monthly unit and included as a covariate. The study period comprised 12 consecutive months, with six months in the pre-intervention phase and six months in the post-intervention phase; the time variable was coded sequentially from 1 to 12 to represent calendar months. Monthly hospital admissions were included as an offset variable to model event rates per admission, and adjusted rate ratios with 95% confidence intervals (CIs) were reported. In addition, a logistic regression model was used to further assess the association between RRT implementation and in-hospital mortality.

To evaluate temporal changes in the intervention effect, an interrupted time series (ITS) analysis was performed using segmented regression. The ITS model included terms for the baseline time trend, the immediate level change associated with RRT implementation (intervention), and the post-intervention change in monthly trend (time-intervention interaction). Seasonality was not explicitly modelled, as the pre- and post-intervention periods were deliberately matched to equivalent calendar months (November to April in consecutive years) to minimise seasonal confounding. Given the short duration of the study and alignment of calendar periods, additional seasonal terms were not included.

A p-value of less than 0.05 was considered statistically significant for all analyses. Statistical analyses were conducted using R statistical software (R Foundation for Statistical Computing, Vienna, Austria, https://www.R-project.org/).

## Results

A total of 24,568 hospital admissions were recorded during the study period, with 11,150 admissions in the pre-RRT phase (November 2022 to April 2023) and 13,418 in the post-RRT phase (November 2023 to April 2024).

**Figure 1:**
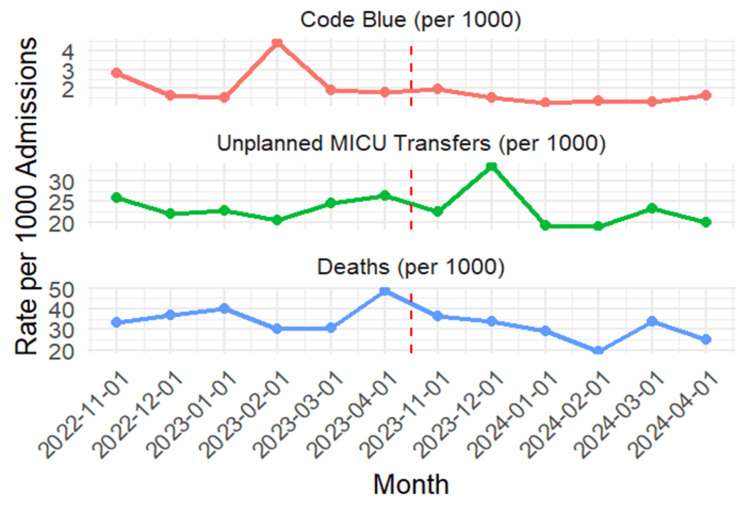
Monthly trends in Code Blue events, MICU transfers, and mortality Vertical dashed line indicates RRT implementation (April 2023) MICU: Medical Intensive Care Unit; RRT: Rapid Response Team

Table 2 summarizes the descriptive comparison of hospital outcomes between the two periods. While overall admissions increased, the rate of unplanned MICU transfers per 1,000 admissions showed a slight decrease (23.6 vs. 22.7), Code Blue events reduced from 2.33 to 1.49 per 1,000 admissions, and in-hospital mortality declined from 3.65% to 2.94%. Figure 1 further illustrates monthly trends in Code Blue events, unplanned MICU transfers, and in-hospital deaths per 1,000 admissions. Post RRT, a sustained reduction in code blue rates and mortality is observed, along with a stabilization of unplanned MICU transfers despite some month-to-month variability.

**Table 2:** Hospital outcomes before and after RRT implementation *Rates per 1,000 hospital admissions and mortality rates (%) were calculated using total hospital admissions as the denominator for each respective time period. Pre-RRT study period: November 2022 – April 2023; Post-RRT study period: November 2023 – April 2024 MICU: Medical Intensive Care Unit; RRT: Rapid Response Team

Outcome Measure	Pre-RRT Period (admissions=11,150)	Post-RRT Period (admissions=13,418)
Code Blue Events		
Number of events	26	20
Events per 1,000 admissions	2.33	1.49
Unplanned Transfers to MICU		
Number of transfers	264	305
Transfers per 1,000 admissions	23.6	22.7
In-hospital Mortality		
Number of deaths	407	395
Mortality rate (%)	3.7	2.9

Demographic data were available only for patients experiencing Code Blue events and not for in-hospital deaths or unplanned transfers. The demographic profile of patients experiencing Code Blue events was comparable between the pre- and post-RRT periods. The mean age was 74.5 ± 8.7 years pre-RRT and 74.9 ± 10.6 years post-RRT, indicating that both groups had a similar age distribution. Female patients accounted for 46.2% of Code Blue events in the pre-RRT period and 40.0% in the post-RRT period, suggesting a slight but non-significant variation in sex distribution. These findings imply that the observed outcomes are unlikely to be confounded by major demographic differences between the two groups.

Table 3 compares event rates before and after RRT implementation using Poisson rate tests. The rate of Code Blue events was lower post RRT (rate ratio (RR): 0.64, 95% CI: 0.34-1.19), but this reduction was not statistically significant (p = 0.14). Similarly, unplanned MICU transfer rates showed no significant change (RR: 0.96, 95% CI: 0.81-1.14; p = 0.64). In contrast, the rate of in-hospital deaths was significantly reduced in the post-RRT period (RR: 0.81, 95% CI: 0.70-0.93; p = 0.0025), suggesting a 19% relative reduction in mortality associated with RRT implementation. While the Poisson test for mortality measured the relative rate of deaths per hospital admission, we also performed a two-proportion test to evaluate the absolute difference in mortality percentages, which confirmed a significant reduction of 0.71% (from 3.65% to 2.94%, p = 0.0019).

**Table 3:** Comparison of rates before and after RRT implementation *Event rates were compared using Poisson rate tests, with hospital admissions as the exposure time. Mortality was also evaluated using a two-proportion test MICU: Medical Intensive Care Unit; RRT: Rapid Response Team

Outcome	Rate Ratio (Post RRT vs. Pre RRT)	95% CI	p-value
Code Blue events	0.64	0.34 – 1.19	0.14
MICU transfers	0.96	0.81 – 1.14	0.64
In-hospital deaths	0.81	0.70 – 0.93	0.0025

**Figure 2:**
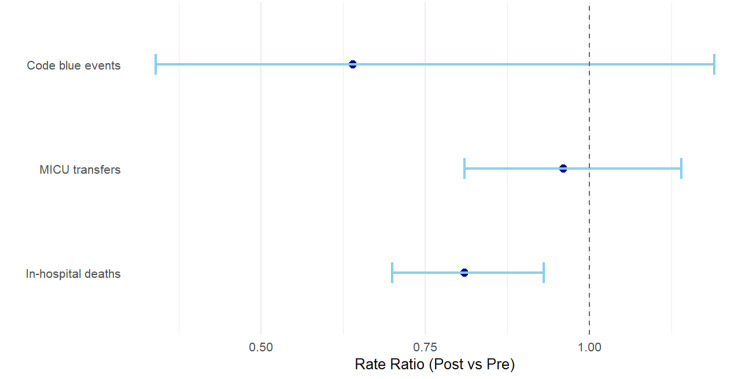
Forest plot for Code Blue events, MICU transfers, and in-hospital deaths pre- and post-RRT implementation MICU: Medical Intensive Care Unit; RRT: Rapid Response Team

Figure 2 illustrates the rate ratios (RRs) and 95% CIs for code blue events, unplanned MICU transfers, and in-hospital mortality before and after RRT implementation. While the plot shows a trend toward fewer code blue events (RR = 0.64) and minimal change in unplanned MICU transfers (RR = 0.96), these effects are not statistically significant as their confidence intervals cross 1. In contrast, the mortality RR (0.81) lies entirely below 1, visually reinforcing the statistically significant reduction in in-hospital deaths observed in Table 3.

Table 4 presents adjusted RRs for Code Blue events, MICU transfers, and in-hospital deaths before and after RRT implementation, accounting for hospital admissions and month-to-month trends. Across all outcomes, the intervention effect was not statistically significant, with RRs close to 1 (e.g., in-hospital deaths: RR: 0.88, 95% CI: 0.66-1.17, p = 0.37). While the point estimates for Code Blue events and mortality (RR < 1) suggest a potential reduction in adverse events, the wide confidence intervals indicate uncertainty, likely due to the limited sample size and short six-month pre- and post-intervention periods. No significant month-to-month trends were observed, suggesting that event rates were relatively stable during the study period. A logistic regression model of in-hospital mortality showed similar findings, with an OR of 0.87 (95% CI: 0.65-1.17), indicating no significant difference in mortality before and after RRT implementation.

**Table 4:** Adjusted rate ratios for Code Blue events, MICU transfers, and in-hospital deaths *Rate ratios were derived from Poisson regression models with hospital admissions included as an offset and controlled for time. MICU: Medical Intensive Care Unit

Outcome	Predictor	Rate Ratio (95% CI)	p-value
Code Blue events	Intervention	0.73 (0.22–2.42)	0.61
	Time (per month)	0.98 (0.82–1.16)	0.8
MICU transfers	Intervention	1.11 (0.79–1.55)	0.56
	Time (per month)	0.98 (0.93–1.03)	0.35
In-hospital deaths	Intervention	0.88 (0.66–1.17)	0.37
	Time (per month)	0.99 (0.95–1.03)	0.51

**Figure 3:**
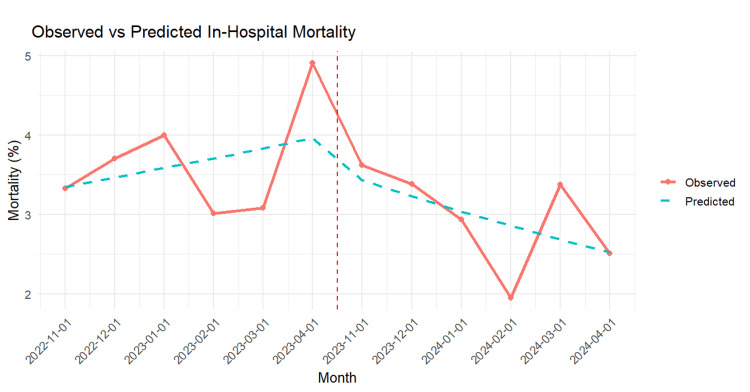
Interrupted time series analysis of in-hospital mortality rates Dashed, red line indicate rapid response team implementation

To further explore temporal patterns beyond the overall pre- vs. post-RRT comparison, we conducted an interrupted time series (ITS) analysis for in-hospital mortality (Table 5). While the logistic regression model showed no significant immediate change in mortality following RRT implementation (OR = 0.92, p = 0.56), the ITS model revealed a significant post-intervention trend, with monthly odds of mortality declining by approximately 9% per month (PostTime OR: 0.91, 95% CI: 0.83-0.99, p = 0.021). This suggests that the RRT effect was not instantaneous but rather evolved over time as the team became integrated into hospital workflows. The ITS plot (Figure 3) supports this finding, showing a downward trend in predicted mortality rates following the RRT launch.

**Table 5:** Interrupted time series (ITS) analysis for in-hospital mortality

Variable	OR (95% CI)	p-value
Time	1.04 (0.98 – 1.10)	0.25
Intervention	0.92 (0.69 – 1.22)	0.56
Post*Time	0.91 (0.83 – 0.99)	0.021

## Discussion

The implementation of an RRT in our tertiary care teaching hospital was associated with a decline in in-hospital mortality, as well as non-significant reductions in Code Blue events and unplanned ICU transfers. While only the mortality reduction was statistically significant in the unadjusted pre-post analysis, the direction of change across outcomes suggests potential improvements in clinical response and patient safety.

However, these improvements were not sustained in adjusted Poisson regression models that accounted for long-term background trends, suggesting that part of the observed benefit may reflect gradual improvements unrelated to the RRT. Notably, ITS analysis revealed a significant month-on-month decline in in-hospital mortality following RRT implementation. This suggests that the positive impact of the RRT may have accumulated gradually as it became more embedded in routine hospital operations.

The statistically significant reduction in in-hospital mortality observed in our study following the implementation of the RRT aligns with a growing body of evidence supporting the effectiveness of RRTs in improving patient outcomes. Several studies, ranging from observational cohorts to cluster-randomized trials and meta-analyses, have documented similar findings. For instance, Solomon et al. (2016) conducted a comprehensive meta-analysis of 30 studies and found a significant reduction in hospital mortality associated with RRT/MET implementation [11]. Earlier systematic reviews, including one commissioned as part of a broader patient safety strategy, reached similar conclusions. Moderate-strength evidence from a high-quality meta-analysis of 18 studies, complemented by findings from 26 additional before-and-after studies, suggested that RRTs not only reduce arrest rates outside the ICU but are also associated with improved overall survival [3]. Longitudinal real-world studies have reinforced this. Beitler et al. (2011), in a long-term cohort study in a large urban academic hospital, reported a sustained decline in hospital-wide mortality following the introduction of an RRT, highlighting the long-term potential of such systems when effectively implemented [14]. Together, these findings situate our study within a robust and evolving body of literature, reinforcing the view that RRTs, particularly when supported by institutional commitment, training, and continuous refinement, can serve as a meaningful tool in improving hospital-wide outcomes.

The observed decline in in-hospital mortality following the implementation of the RRT in our hospital is likely attributable to several interrelated clinical and organizational mechanisms that align with theoretical models and empirical findings from existing literature. Prior to RRT implementation, clinical deterioration in non-code patients often went unrecognized or was escalated in an ad hoc, unstructured manner. Decision-making frequently relied on the individual judgment of junior staff, often leading to delays in intervention and ICU transfer. This "failure to rescue" dynamic is well documented and represents a critical patient safety concern in acute care hospitals [16,17]. The RRT addressed this by introducing a formalized escalation pathway, empowering bedside nurses and junior clinicians to initiate a team-based response based on predefined physiological triggers. This early warning paradigm is consistent with the conceptual framework of “afferent limb” activation described by DeVita et al. (2006), which emphasizes timely recognition of deterioration as a key determinant of RRT effectiveness [4].

In our setting, the most common reason for RRT activation was acute desaturation (SpO₂ < 90%), which accounted for 66% of calls, followed by abnormalities in heart rate and respiratory rate. These activation patterns suggest that the RRT was frequently mobilized in response to early signs of respiratory compromise, a finding consistent with other studies that highlight hypoxia as a leading cause of in-hospital deterioration [9,18]. This pattern underscores the RRT’s role in providing timely respiratory assessment and intervention at the bedside, potentially preventing further decompensation and avoiding full cardiac arrest. This pattern underscores the RRT’s role in providing timely respiratory assessment and intervention at the bedside, potentially preventing further decompensation and avoiding full cardiac arrest.

Chan et al. (2010) emphasized that the success of RRTs hinges not only on the presence of a team but also on how well activation protocols are understood and integrated into routine clinical practice [3]. Similarly, Winters et al. (2013) identified several implementation factors that underpin the impact of RRTs: team composition (e.g., physician-led vs nurse-led), the availability of experienced responders, and organizational support mechanisms such as communication infrastructure and ongoing staff education [7]. These components collectively enhance the “efferent limb” of the system-the team’s ability to respond rapidly, assess critically ill patients, and institute timely interventions. Furthermore, RRTs help bridge the traditional divide between ward-level monitoring and ICU-level care, offering advanced assessment and treatment capabilities at the bedside before deterioration becomes irreversible.

By fostering a proactive rather than reactive care environment, RRTs also contribute to a shift in safety culture. This includes greater accountability, improved interdisciplinary communication, and increased vigilance among staff. As noted by DeVita et al. (2006), the cumulative impact of these systems often lies not in a single intervention but in the creation of a structured, reliable process for detecting and responding to early signs of patient decline [4]. In our setting, this was operationalized through standardized activation criteria, a target response time of five minutes, and structured team coordination, all of which likely contributed to the observed improvements in patient outcomes.

Notably, the post-implementation period also saw a rise in total and average monthly hospital admissions. While not the primary focus of our analysis, this increase may reflect enhanced institutional trust in the hospital’s capacity to manage acutely deteriorating patients. Moreover, improved surveillance and early detection mechanisms may have led to the timely admission of higher-risk patients, contributing indirectly to improved outcomes by expanding the clinical window for pre-emptive intervention. These contextual shifts, in tandem with system-level changes, likely contributed to the observed reduction in in-hospital mortality.

While our unadjusted and ITS analyses indicated a statistically significant reduction in in-hospital mortality following RRT implementation, this effect was not sustained in adjusted Poisson regression models that accounted for underlying time trends. This finding diverges from studies such as Beitler et al. (2011), who reported a sustained and significant decline in hospital-wide mortality even after controlling for secular trends over a longer observation period [14]. One possible explanation for this discrepancy lies in the duration and structure of our dataset: with only six months of data pre- and post-intervention, the number of time points available for trend-adjusted models was limited. This likely reduced statistical power and increased the risk of overfitting, thereby attenuating observed effects in more complex models.

ITS models are more robust to small sample sizes when there is a strong post-intervention trend, which may explain why the mortality effect remained statistically significant in our ITS analysis but not in the trend-adjusted regression. Taken together, these findings suggest a possible association between RRT implementation and reduced in-hospital mortality, while underscoring that causal inference is limited and the observed effects should be interpreted cautiously in light of the study design. This highlights an important methodological consideration for future evaluations: adequate temporal sampling is critical when attempting to isolate intervention effects from background variation. As others have noted, detecting the full impact of RRTs may require longer implementation lags and sustained observation periods to account for organizational learning curves and cultural adaptation [7,8].

Despite the observed statistically significant reduction in in-hospital mortality following RRT implementation, our study did not find significant changes in the rates of Code Blue events or unplanned MICU transfers. This pattern, where mortality declines without corresponding reductions in other adverse clinical events, has been observed in several real-world evaluations of RRTs and points to the complex and multifactorial nature of clinical deterioration and rescue in hospitalized patients. For instance, a meta-analysis by Hall et al. (2020) noted that while RRTs are moderately effective at reducing hospital mortality, their impact on ICU transfer rates remains inconclusive, and reductions in cardiac arrest rates are not always statistically significant [8].

One possible explanation is that the mortality benefit in our setting may have stemmed more from improved timing and quality of clinical response rather than a reduction in the occurrence of critical events themselves. In other words, patients may have continued to experience acute deterioration, but the presence of a rapid, structured, and protocol-driven response may have increased the likelihood of survival after these events. Our activation data support this interpretation: desaturation was the most common trigger for RRT calls, suggesting that respiratory compromise was often identified and managed promptly, potentially preventing fatal outcomes even if ICU transfer or Code Blue activation remained necessary. It is also worth noting that, as discussed earlier, the benefits of RRTs may accrue gradually; our relatively short post-implementation window may not have been sufficient to detect meaningful changes in Code Blue events or ICU transfer practices

Finally, the absolute number of events, especially for Code Blues, was relatively small, which may have limited our statistical power to detect significant changes in these outcomes despite downward trends in event rates (e.g., Code Blue events per 1000 admissions fell from 2.33 to 1.49). Prior studies, such as Segon et al. (2014), also reported no statistically significant change in mortality or arrest rates following RRT implementation in small- to mid-sized community-based hospitals, attributing this in part to similar baseline care processes and limited sample sizes [13].

This study’s pre-post design enabled direct comparison of outcomes before and after RRT implementation, with analysis of multiple endpoints, mortality, Code Blue events, and ICU transfers, enhancing its comprehensiveness. Use of ITS modeling added rigor to the assessment of temporal trends. Nevertheless, several limitations should be considered when interpreting these findings. The absence of a concurrent control group and the relatively short follow-up period may have limited causal inference and the ability to detect delayed intervention effects. In addition, patient-level characteristics were unavailable for key outcomes (except for Code Blue events), restricting the extent of risk adjustment. While RRT structure and activation criteria were standardised, detailed process measures, such as activation frequency, response-time adherence, and intervention profiles, were not available for quantitative analysis, limiting assessment of implementation fidelity and underlying mechanisms. Furthermore, deterioration related to adverse drug events or drug-drug interactions was not systematically captured, precluding assessment of their contribution to RRT activation or clinical outcomes. Finally, the single-centre design limits generalisability, and although ICU capacity, staffing ratios, and monitoring systems remained unchanged during the study period, other unmeasured factors-such as evolving clinical practices, increasing staff familiarity with early warning signs, parallel quality improvement initiatives, or changes in escalation culture-may have influenced observed outcomes over time.

Larger, multi-center studies with longer follow-up and stepped-wedge designs are needed to validate these findings. Evaluations of cost-effectiveness, team structure, and escalation protocols would further inform RRT optimization. Improved data capture and hospital information system integration may enhance future monitoring and response efficiency. Taken together, our findings suggest that while RRT implementation can contribute to improved survival, its effect on intermediate outcomes such as ICU transfers and Code Blue events may be variable and context-dependent, shaped by institutional practices, team maturity, and existing escalation norms.

## Conclusions

Implementation of an RRT in this tertiary care hospital was associated with lower in-hospital mortality. No statistically significant changes were seen in Code Blue activations or unplanned ICU transfers. Taken together, these findings suggest a possible benefit of earlier clinical intervention following RRT implementation, while underscoring the limitations of causal inference inherent to a short-duration, single-centre, pre-post study. Further controlled, multicentre studies with longer follow-up and enhanced risk adjustment are warranted to confirm effectiveness and generalisability in similar low-resource, high-burden healthcare settings.

## Appendices

**Table 6:** RAPID RESPONSE TEAM (RRT) ACTIVATION FORM

Patient Details			
Name		Age	
Gender		UHID Number	
Ward/Room Number			
RRT Activation			
Name & Role of Person Activating RRT		Time of RRT Call	: AM/PM
Reason for Activating RRT (Check all that apply)			
Heart rate <40 or >130 bpm	Respiratory rate <10 or >30		
Chest pain/discomfort	Unexpected altered mental status		
SpO2 <90% on oxygen	Urine output <100 ml in 4 hrs		
Systolic BP <90 mmHg	Agitated patient		
New/prolonged seizure	Abnormal bleeding		
Nursing/family concern			
RRT Arrival & Departure			
Time of RRT Arrival	: AM/PM	Time of RRT Departure	: AM/PM
Names and Roles of RRT Members			
Patient Assessment Findings			
BP		PR	
Temp		SpO2	
RR		GRBS	
Interventions Performed by RRT			
Airway/Breathing		Circulation	
Oral airway		Fluid bolus	
Suctioned		Blood	
Nebulizer treatment		EKG	
Intubated		Defibrillation	
NPPV		Cardioversion	
Bag mask		No intervention	
O2 mask/nasal			
Arterial Blood Gas			
Chest X-Ray			
Disposition and Notifications			
Patient Disposition	Remained in unit / Transferred to Medical ICU		
Attending Physician Notified	YES/NO	Code Status Discussed	YES/NO
Plan of Care			
Re-Assessment and Outcome			
Re-Assessment Time	: AM/PM	Patient Outcome in 24 hours	Alive / Deceased
